# Recurrent Atypical Hemolytic-Uremic Syndrome (aHUS) Associated With CD46 Genetic Mutation: A Report of a Rare Case

**DOI:** 10.7759/cureus.103475

**Published:** 2026-02-12

**Authors:** Navanita Biswas, Prakash Adhikari, Nisha Baral

**Affiliations:** 1 Internal Medicine, Tower Health, Reading Hospital, West Reading, USA; 2 Microbiology, Manipal College of Medical Sciences, Pokhara, NPL

**Keywords:** ahus, eculizumab, genetic mutation, plasma exchange therapy, recurrent ahus

## Abstract

Hemolytic-uremic syndrome (HUS) is a thrombotic microangiopathy (TMA) characterized by microangiopathic hemolytic anemia, thrombocytopenia, and renal impairment. The typical form of HUS is most often associated with Shiga toxin-producing *Escherichia coli* infection, whereas atypical HUS (aHUS) is a rare variant caused by genetic mutations that disrupt complement regulation. This dysregulation promotes complement deposition on vascular endothelium, leading to microangiopathic hemolysis, platelet consumption, and organ injury, with acute kidney injury being the most common clinical manifestation. We present the case of a 38-year-old male who presented with nonspecific symptoms and was found to have thrombocytopenia, acute kidney injury, and intravascular hemolysis. Laboratory tests showed a negative direct Coombs test, normal ADAMTS13 activity, and a normal bone marrow biopsy. Although a kidney biopsy was considered, it was avoided due to thrombocytopenia, and the diagnosis was made on clinical and laboratory grounds. Plasma exchange and methylprednisolone were initiated but did not improve his platelet count or renal function. He was subsequently started on eculizumab, a monoclonal antibody against complement, which resulted in significant clinical and laboratory improvement. This case report highlights the importance of early diagnosis and appropriate treatment to prevent morbidity and mortality from aHUS. Although it is a rare condition, clinicians should maintain a high index of suspicion for aHUS in patients presenting with features of TMA.

## Introduction

Thrombotic microangiopathies (TMAs) encompass a spectrum of disorders, including thrombotic thrombocytopenic purpura (TTP) and hemolytic-uremic syndrome (HUS). The typical features of TMAs include the triad of microangiopathic hemolytic anemia, thrombocytopenia, and organ injury, which result from endothelial damage and microvascular thrombosis [1].

HUS is further classified into infection-associated HUS (commonly due to Shiga toxin-producing *Escherichia coli*, serotype O157:H7), secondary HUS associated with coexisting medical conditions such as autoimmune diseases, infections, transplantation, malignancies, cytotoxic drugs, or pregnancy, cobalamin C defect-associated HUS, and atypical HUS (aHUS), which is linked to mutations in genes coding for complement regulators, particularly complement factor H (CFH) [2]. Mutations in other complement regulators and proteins, including complement component 3 (C3), factor B, factor I, and CD46, can also cause this disorder [3].

aHUS is rare, with an estimated incidence of 0.23 to 0.42 cases per million population per year. Although often described in pediatric populations, registry data show that many cases present in adulthood, with variable clinical expression depending on the underlying genetic mutation. Both sexes appear to be affected, with no strong gender predilection [4]. It generally has poor clinical outcomes due to a higher risk of morbidity and mortality compared with typical HUS. Timely diagnosis and early initiation of appropriate treatment can minimize complications and reduce mortality. Current therapies include therapeutic plasma exchange and eculizumab, a monoclonal antibody targeting complement component C5 [5].

## Case presentation

A 38-year-old male with a history of hypothyroidism presented to the ED with generalized body aches, flu-like symptoms, flank pain, and dark urine for one week. He denied any recent diarrheal illness. There was no family history of autoimmune or hematologic disorders, and his social history was unremarkable.

On examination, vital signs were a temperature of 98.6°F, a heart rate of 104 bpm, a blood pressure of 152/94 mmHg, a respiratory rate of 20/min, and an oxygen saturation of 99% on room air. Physical examination revealed pallor; no rash, arthritis, or neurologic deficits were noted.

Laboratory investigations revealed thrombocytopenia, acute kidney injury, indirect hyperbilirubinemia, elevated lactate dehydrogenase, and low haptoglobin. Complement studies showed decreased C3 and C4 levels. Glucose-6-phosphate dehydrogenase (G6PD) level was normal. Workup for hemophagocytic lymphohistiocytosis (HLH), autoimmune disease, and infections was negative, as summarized in Table 1, Table 2, and Table 3. A peripheral blood smear demonstrated rare schistocytes. Although the original smear image was not archived, the hematology team confirmed the presence of schistocytes consistent with microangiopathic hemolytic anemia.

**Table 1:** Complete blood count at presentation

Parameter	Patient value	Reference range	Units
White blood cells	5	4.8-10.8	10³/μL
Red blood cells	3.98	4.50-6.10	10⁶/μL
Hemoglobin	12.8	14.0-17.5	g/dL
Hematocrit	35.5	39-58	%
Mean corpuscular volume	89.9	80-99	fL
Mean corpuscular hemoglobin	32.2	27-34	pg
Mean corpuscular hemoglobin concentration	35.8	31-37	g/dL
Red cell distribution width	12.2	11-16	%
Platelets	6	130-400	10³/μL

**Table 2:** Comprehensive metabolic panel

Test	Result	Reference range	Units
Sodium	133	135-145	mmol/L
Potassium	3.5	3.5-5.0	mmol/L
Chloride	99	98-106	mmol/L
CO₂ (bicarbonate)	24.1	22-29	mmol/L
Anion gap	10	8-16	mmol/L
Blood urea nitrogen	29	7-20	mg/dL
Creatinine	3.74	0.6-1.3	mg/dL
Glucose (fasting)	130	70-99	mg/dL
Calcium	9.2	8.5-10.5	mg/dL
Aspartate aminotransferase	65	10-40	IU/L
Alanine aminotransferase	17	7-56	IU/L
Direct bilirubin	0.3	0.0-0.3	mg/dL
Total bilirubin	2.6	0.1-1.2	mg/dL
Lactate dehydrogenase	1109	140-280	IU/L

**Table 3:** Additional laboratory tests

Test	Result	Reference range	Units
Direct Coombs test	Negative	Negative	-
ADAMTS-13 activity	93	>5	%
D-dimer	3.49	<0.53	µg/mL
Haptoglobin	<30	30-200	mg/dL
Ferritin	>7500	27-300	ng/mL
Fibrinogen	196	175	mg/dL
Triglycerides	256	193	mg/dL

Abdominal and pelvic imaging did not reveal splenomegaly. The differential diagnoses considered included TTP, HUS, idiopathic thrombocytopenic purpura, autoimmune hemolytic anemia, G6PD deficiency, and HLH.

Hematology and nephrology were consulted, and the patient was started on intravenous methylprednisolone and plasma exchange. After five sessions of plasma exchange, there was no improvement in his platelet count. The direct Coombs test was negative, ADAMTS13 activity was normal, and bone marrow biopsy showed normal cellularity, effectively excluding autoimmune hemolytic anemia, TTP, and hematologic malignancy. The diagnosis was therefore considered more consistent with aHUS. Given the inadequate response, therapy was escalated to eculizumab after meningococcal vaccination and antibiotic prophylaxis. His platelet count improved significantly the following day, and his kidney function continued to recover. He was subsequently discharged home.

Five years later, he again presented to the ED with similar symptoms, including hematuria and a flu-like illness. He was found to have hemolytic anemia, acute kidney injury, and thrombocytopenia. He received a session of plasma exchange, followed by treatment with eculizumab. His platelet count improved the following day, and he was discharged home with outpatient hematology follow-up. After the induction dose of eculizumab, he was scheduled to receive maintenance doses every eight weeks for a total of three doses.

During this second admission, genetic testing using the Machaon Diagnostics aHUS Complement Panel, via next-generation sequencing, revealed a heterozygous stop-gain (nonsense) variant in the CD46 gene, classified as pathogenic according to the American College of Medical Genetics and Genomics criteria [6] and associated with aHUS. This pathogenic variant leads to premature protein truncation, impairing membrane cofactor protein (MCP) function, a known contributor to dysregulated complement activation in aHUS.

## Discussion

aHUS is caused by sporadic or genetic insults that lead to dysfunction of the complement cascade, resulting in complement deposition on endothelial cells, endothelial swelling, and capillary thickening. This process causes protein and cellular infiltration, inducing a prothrombotic state with the formation of platelet-rich thrombi in arterioles and capillaries, leading to partial or complete vessel occlusion and subsequent tissue ischemia [5,7].

The most frequently implicated gene is CFH, but pathogenic variants have also been identified in complement factor I (CFI), MCP (CD46), C3, complement factor B (CFB), and thrombomodulin. Additionally, recessive mutations in diacylglycerol kinase epsilon have been described, particularly in pediatric cases [8]. In our patient, genetic testing revealed a stop-gain variant in CD46. This variant results in premature protein termination, leading to loss of MCP function and impaired regulation of complement activation.

The clinical presentation of aHUS typically includes features of hemolytic anemia, thrombocytopenia, and impaired renal function, depending on the extent of microvascular injury, thrombosis, and tissue ischemia [5]. Triggers for aHUS include nonenteric bacterial and viral infections, medications, malignancies, transplantation, pregnancy, and other underlying medical conditions; however, in more than 30% of cases, the trigger remains unknown [9].

For the identification of aHUS, the initial step is to establish the diagnosis of TMA based on typical clinical features and laboratory findings, including microangiopathic hemolysis, thrombocytopenia, and organ damage, such as renal impairment or neurological or gastrointestinal symptoms. Subsequently, it is essential to differentiate among Shiga toxin-associated HUS, TTP, and aHUS. In our patient, there was no history of diarrhea or bloody stools, making Shiga toxin-associated HUS unlikely. TTP was ruled out based on normal ADAMTS13 activity. Differentiating aHUS from secondary HUS can be challenging. In our patient, common causes of secondary HUS were excluded through evaluation for autoimmune conditions and a bone marrow biopsy, which was negative for hematologic malignancy.

Complications of aHUS include severe thrombocytopenia, anemia, bleeding, and acute kidney injury. Extrarenal manifestations occur in approximately 20% of patients and can involve multiple organ systems, including cardiovascular (cardiomyopathy and myocardial infarction), peripheral vascular (digital gangrene), pulmonary (pulmonary hemorrhage), gastrointestinal (bleeding and pancreatitis), and musculoskeletal (rhabdomyolysis) systems [10].

Treatment of aHUS begins with supportive care, including fluid and electrolyte management and transfusion of packed red blood cells as needed. Platelet transfusion is generally avoided due to the risk of exacerbating microvascular thrombosis and is reserved only for cases of active bleeding or prior to invasive procedures. Specific therapies, such as plasma exchange and eculizumab, are often required to achieve remission. Empiric plasma exchange is generally initiated after the initial diagnosis of aHUS, as it helps remove antibodies and other pathogenic proteins [5]. In settings where complement inhibitors are not available, plasma exchange remains the standard of care; however, it does not address the underlying complement dysregulation. In our patient, plasma exchange did not result in significant clinical improvement.

Eculizumab is a humanized monoclonal IgG antibody that binds to complement protein C5, preventing its cleavage into C5a and C5b. It has been shown to induce remission in acute episodes of aHUS refractory to plasma therapy. Eculizumab should be considered first-line therapy when the diagnosis of aHUS is uncertain or in patients with mutations in CFH, CFI, CFB, or C3 [11]. Several studies have demonstrated improved outcomes with early initiation of eculizumab, including better renal recovery and normalization of platelet counts [12]. The optimal duration of eculizumab treatment has not been established; however, one study reported a median treatment duration of 90 days to achieve remission [13].

Other therapeutic agents are under investigation, including ravulizumab, a long-acting C5 inhibitor with less frequent dosing, and iptacopan, an oral factor B inhibitor currently studied in the APPLE-HUS trial. These agents may improve long-term disease management and reduce treatment burden. Important considerations include vaccination and prophylaxis against *Neisseria meningitidis *and other encapsulated organisms prior to eculizumab therapy due to increased infection risk.

## Conclusions

aHUS is a rare condition associated with high morbidity and mortality if not diagnosed and treated promptly. Patients often present with nonspecific symptoms, and laboratory findings consistent with TMA should prompt urgent evaluation. Contemporary literature demonstrates improved renal and hematologic outcomes with early complement inhibition therapy, particularly in genetically confirmed cases. Our case adds to existing reports of CD46-associated aHUS by highlighting recurrence after a prolonged remission and rapid response to eculizumab, emphasizing the importance of long-term hematology follow-up and genetic counseling. Early recognition, structured diagnostic evaluation, and timely initiation of complement-targeted therapy remain critical to optimizing prognosis.
